# The congenital melanocytic nevus: a rare clinical image

**DOI:** 10.11604/pamj.2022.42.250.35727

**Published:** 2022-08-03

**Authors:** Darshana Kumari, Tejaswee Lohakare

**Affiliations:** 1 Department of Child Health Nursing, Smt. Radhikabai Meghe Memorial College of Nursing, Datta Meghe Institute of Medical Sciences, Sawangi (Meghe), Wardha, Maharashtra, India

**Keywords:** Congenital melanocytic nevus, paediatric cancer, dark pigmentation, hair growth

## Image in medicine

Congenital melanocytic nevus affects around 1% of newborns. Facial melanocytic nevus may be linked to an increased risk of paediatric cancer. Furthermore, because the lesions reveal hair and dark pigmentation, the cosmetic and emotional impacts might be substantial. A 6-year-old female was brought to the outpatient department with complaints of black discoloration of the left side of the face and excessive hair growth on a particular site. After detailed history collection and physical examination, it reveals that the black discoloration is since birth. The physician diagnosed her with congenital melanocytic nevus and hence referred them to the dermatology department for further management.

**Figure 1:**
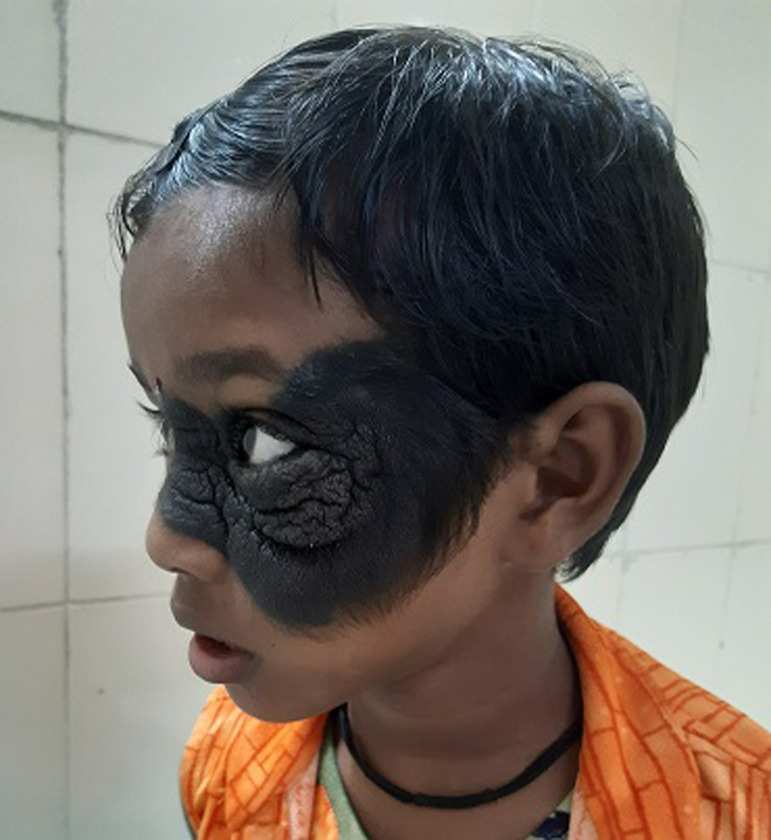
black pigmentation on left side of face with excessive hair

